# Smoking is associated with ulnar nerve entrapment: a birth cohort study

**DOI:** 10.1038/s41598-019-45675-1

**Published:** 2019-07-01

**Authors:** Sina Hulkkonen, Juha Auvinen, Jouko Miettunen, Jaro Karppinen, Jorma Ryhänen

**Affiliations:** 1 Department of Hand Surgery, Helsinki University Hospital and University of Helsinki, Helsinki, Finland; 2 Center for Life Course Health Research, University of Oulu, Oulu, Finland; 3 Medical Research Center Oulu, Oulu University Hospital and University of Oulu, Oulu, Finland; 4 Oulunkaari Health Center, Ii, Pudasjärvi, Finland; 5 Finnish Institute of Occupational Health, Oulu, Finland

**Keywords:** Epidemiology, Risk factors

## Abstract

Ulnar nerve entrapment is the second most common compression neuropathy of the upper extremity. It has been associated with smoking in cross-sectional studies. Our aim was to study whether smoking is associated with ulnar nerve entrapment. The study population consisted of the Northern Finland Birth Cohort 1966 participants, who attended the 31-year follow-up in 1997 (N = 8,716). Information on smoking, body mass index (BMI), long-term illnesses, and socio-economic status were recorded at baseline in 1997. Data on hospitalizations due to ulnar nerve entrapment neuropathies was obtained from the Care Register for Health Care, 1997–2016. Hazard ratios (HR) with 95% confidence intervals (CI) and population attributable risk (PAR) were calculated adjusted for gender, BMI and socio-economic status. 66 patients were diagnosed with ulnar nerve entrapment in the follow-up 1997–2016. Before the age of 31 years, smoking ≤10 pack years associated with more than doubled (HR = 2.57, 95% CI = 1.29–5.15) and smoking >10 pack years with more than five-folded (HR = 5.61, 95% CI = 2.80–11.23) risk for ulnar nerve entrapment compared to non-smokers in the adjusted analyses. Adjusted PAR for smoking (reference of no smoking) was 53.6%. In our study, smoking associated with increased risk for ulnar nerve entrapment, accounting for considerable proportion of increased risk.

## Introduction

Ulnar nerve entrapment is the second most common compression neuropathy of the upper extremity after carpal tunnel syndrome^1^. The ulnar nerve usually becomes compressed at the elbow, a state called cubital tunnel syndrome. Its prevalence is 0.6–0.8%, and the yearly incidence is 25.2–32.7/100,000 among men and 17.2–18.9/100,000 among women^2–4^. Compression in the Guyon’s canal at the wrist is rarer; its prevalence remains unknown in the general population.

The etiology of ulnar nerve entrapment is considered multifactorial. Recognized risk factors for ulnar nerve entrapment include male gender^3–5^, both low and high body mass index (BMI)^5,6^, systemic disorders such as diabetes^7^, hypothyroidism^8^, renal disease^9^, and occupational biomechanical exposures^10^. Non-operative treatment usually involves physiotherapy and ergonomic adjustments^11^. If such treatment fails or if the entrapment is moderate or severe, surgical treatment might be needed^1^. However, ulnar nerve entrapment neuropathies cause a significant amount of sick leave and healthcare costs^12^.

Current smoking is associated with cubital tunnel syndrome^10,13–15^, and a higher exposure is associated with an increased risk^16,17^. Still, we found no population-based studies about smoking and any ulnar nerve entrapment neuropathies.

The hypotheses of this study were: 1) smoking is associated with an increased risk for ulnar nerve entrapment, and 2) an increase in the amount of tobacco consumed is associated with an increased risk for ulnar nerve entrapment. In this study, all entrapment sites of the ulnar nerve are referred as ulnar nerve entrapment.

## Materials and Methods

### Study population

**Figure 1:**
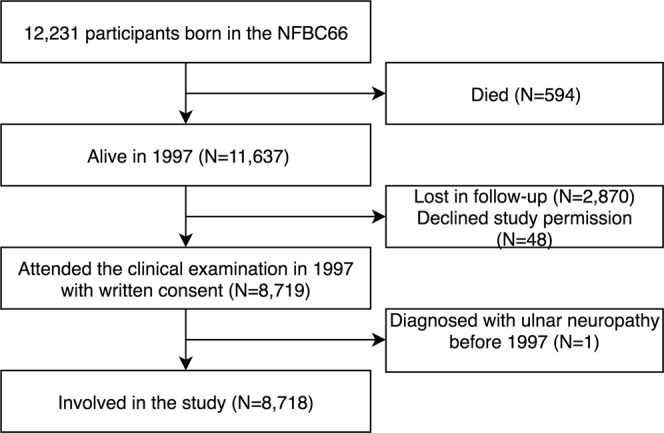
Flowchart of the study population (the Northern Finland Birth Cohort 1966, NFBC66).

The study population consisted of the Northern Finland Birth Cohort 1966 (NFBC1966). It originally included 12,231 children with expected birth dates in 1966 in the Oulu and Lapland provinces^18^. In 1997, when the cohort population turned 31 years, a total of 8,719 cohort participants attended a clinical examination and gave their informed consent to voluntarily participate in the study. One participant was diagnosed with ulnar nerve entrapment before the age of 31 and thus excluded from further analysis. Finally, 8,718 participants were included in the study (Fig. 1).

The participants’ personal identification number was replaced with study identification codes in the study data. The study was approved by the Ethics Committee of the Northern Ostrobothnia Hospital District and followed the principles of the Declaration of Helsinki of the World Medical Association.

### Study population at baseline

The study population had clinical examination in 1997, when the participants were 31 years old. Data was collected via postal questionnaire and at a clinical examination. If the question: “Have you ever smoked in your life?” was answered “Yes”, the questions on smoking habits continued with “Have you ever smoked regularly in your life?”, “At what age did you start smoking?” and “At what age did you quit smoking?” The amount of consumed tobacco products was recorded. Three variables describe smoking before the age of 31 years: regular smoking (no/yes), pack years, and pack years divided into three categories (non-smokers, smokers with a history of 10 or fewer pack years, and smokers with over 10 pack years). Pack years were calculated by multiplying regular smoking years with the amount of tobacco consumed, assuming that one pack contains 20 cigarettes, cigarillos or pipefuls. One cigar was assumed to equate to four cigarettes^19^.

Socio-economic status was defined by occupation and activity in working life with nine categories: farmers, entrepreneurs, clerical workers (lower and upper), manual workers, students, pensioners, the unemployed, and unknown according to Statistics Finland^20^. The categorical variable was formed combining similar socioeconomic groups: clerical workers and entrepreneurs; farmers and manual workers; and students, pensioners and the unemployed. Socio-economic status coded as unknown was handled as missing information. BMI was calculated of height and weight measurements in a clinical examination or, if missing, the height and weight information from the postal questionnaire. The variable was given two categories: underweight/normal (BMI 25 kg/m^2^ or lower) and overweight/obese (BMI over 25 kg/m^2^). Information on long-term illnesses such as diabetes, thyroid diseases and rheumatoid arthritis was collected.

### Study outcome

The study outcome were new cases of diagnosed ulnar entrapment neuropathy occurring after the baseline examination in 1997 until the end of 2016. The data on hospitalizations due to ulnar nerve entrapment were obtained from the Care Register for Health Care. It is a national register covering both public and private hospitals in Finland, containing information about patients’ demographic features, primary and subsidiary diagnoses, surgical procedures, and dates of admission and discharge, including information from outpatient clinics. In Finland, the ulnar neuropathies are diagnosed by clinical examination and measurement of nerve conduction velocity. The diagnoses are coded according to the International Classification of Diagnoses (ICD). The diagnosis of ulnar nerve entrapment was coded 357.3 according to the eighth revision of ICD from 1981 to 1986, 354.2 according to the ninth revision of ICD from 1987 to 1995, and G56.2 according to the tenth revision of ICD from 1996 to 2016. All ulnar entrapment neuropathies are coded under the same code in ICD. The diagnoses were obtained from hospital data, which includes both outpatient- and inpatient-based services in specialist care.

### Statistical analysis

Fisher’s exact and Chi-square tests, and T-tests for normally distributed and Mann-Whitney U-test for non-normally distributed continuous variables were used to examine the correlations between ulnar entrapment neuropathies and the background factors. We used Cox’s proportional hazards regression with 95 percent confidence intervals (CI) to further investigate the role of the background factors in ulnar entrapment neuropathies with various models. In additional analyses, we tested for statistical interactions between smoking and other variables on prediction of ulnar nerve entrapment by adding smoking multiplied by the variable of interest in the multivariate models. To visualize the outcomes (new cases of ulnar entrapment neuropathy) in different groups, Kaplan-Meier curves were added. Population attributable risk percentages (PAR%) were calculated for smoking (with a target value of no smoking) to investigate the role of smoking in patients diagnosed with ulnar nerve entrapment neuropathies. PAR% were adjusted for the same background factors as Cox’s models. Because of the small number of cases, the analyses were not stratified but adjusted by gender. Cases with missing values were not included in the analysis.

### Ethical approval and informed consent

All the study participants gave their informed consent to voluntarily participate in the study. The study was approved by the Ethics Committee of the Northern Ostrobothnia Hospital District and followed the principles of the Declaration of Helsinki of the World Medical Association.

## Results

**Figure 2:**
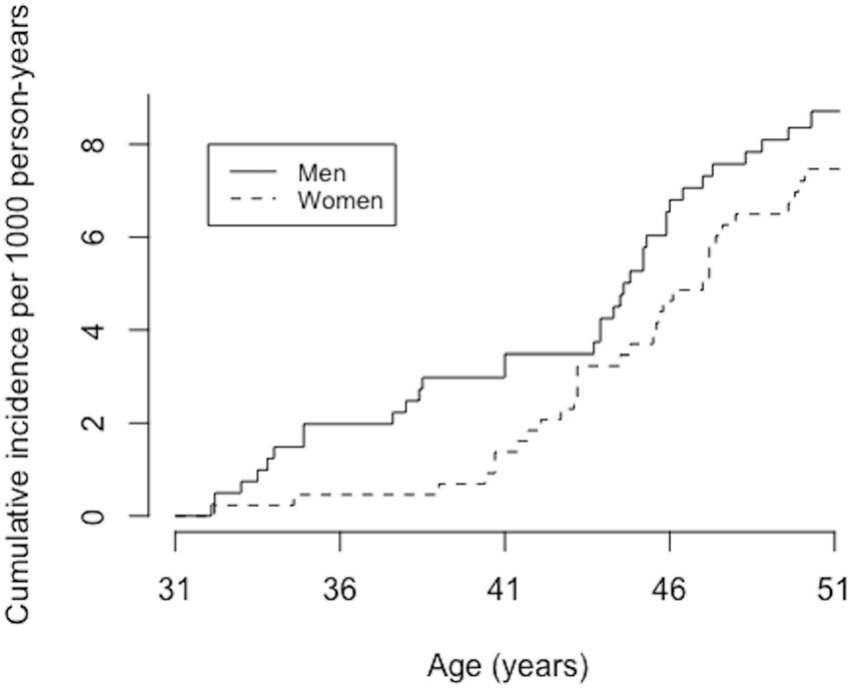
Cumulative incidence of ulnar neuropathy in NFBC66.

The cumulative incidence of ulnar neuropathy in the cohort population (N = 8718) is shown in Fig. 2. Mean follow-up time was 18.48 years (standard deviation, sd = 3.98). During the follow-up from 1997 to 2016, a total of 66 participants were diagnosed with ulnar nerve entrapment. Of those, 34 were men and 32 were women, of whom only 14 were non-smoking. The pack-years for men were higher than for women, 5.33 (sd = 7.40) and 2.41 (sd = 4.55), respectively (P < 0.001). The basic demographic features stratified by smoking are represented in Table 1.

**Table 1:** Basic demographic features of the study population stratified by history of regular smoking.

	Non-smokers, n = 4211	Smokers, n = 4194	P-value	Missing, n (%)
Gender			<0.001	0 (0.0)
Male	1757 (41.7)	2247 (53.6)		
Female	2454 (58.3)	1947 (46.4)		
At the age of 31 years				
History of regular smoking, pack years			<0.001	1252 (14.4)
Mean (sd)	0.0 (0.0)	8.7 (6.8)		
No, n (%)	4211 (100.0)	0 (0.0)		
10 or fewer, n (%)	0 (0.0)	2082 (64.0)		
Over 10, n (%)	0 (0.0)	1173 (36.0)		
BMI			<0.001	316 (3.6)
Mean (sd)	24.3 (4.1)	24.8 (4.2)		
Normal, n(%)	2647 (63.7)	2414 (58.3)		
Overweight/Obese, n(%)	1508 (36.3)	1729 (41.7)		
Socio-economic status, n (%)			<0.001	515 (5.9)
Clerical workers, entrepreneurs	2636 (65.1)	1924 (47.2)		
Students, retired, unemployed	514 (12.7)	678 (16.7)		
Manual workers, farmers	897 (22.2)	1470 (36.1)		
Diabetes, n (%)	56 (1.3)	53 (1.3)	NS	286 (3.3)
Thyroid diseases, n (%)	80 (1.9)	81 (1.9)	NS	284 (3.3)
Rheumatoid arthritis, n (%)	41 (1.0)	45 (1.1)	NS	289 (3.3)
In the follow-up				
Ulnar nerve entrapment*, n (%)	14 (0.3)	49 (1.2)	<0.001	0 (0.0)
NS, not significant				

Of those 66 participants, who were diagnosed with ulnar nerve entrapment during the follow-up, three had hypothyroidism and none had diabetes mellitus, rheumatoid arthritis, renal diseases or cancer at the age of 31 (the study baseline); none of them were diagnosed with them during the follow-up. No correlation between these conditions and ulnar entrapment neuropathy was found in this birth cohort. Ulnar nerve entrapment correlated with smoking (P < 0.001) and socio-economic status (P = 0.026), but not with gender (P = 0.625) or BMI (P = 0.249).

**Figure 3:**
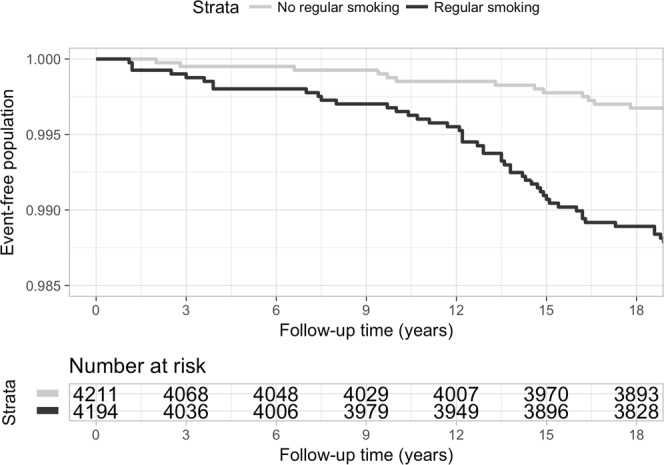
Kaplan-Meier curve of ulnar neuropathy in the study population in follow-up 1997–2016, stratified by history of regular smoking at baseline.

Multiple Cox’s proportional hazard regression models were tested (Table 2). Adjusted for all the background factors, smoking ten or fewer pack years before the age of 31 years was associated with more than doubled (hazard ratio, HR = 2.57, 95% CI = 1.29–5.15) and smoking over ten pack years before the age of 31 years was associated with over five times higher risk (HR = 5.61, 95% CI = 2.80–11.23) for ulnar nerve entrapment later in life compared to non-smokers. The background factors did not associate statistically significantly with ulnar nerve entrapment. In the interaction analysis, none of the variables included in the models had significant interaction with smoking in prediction of ulnar nerve entrapment. Figure 3 illustrates the diagnosed ulnar entrapment neuropathies occurring in study participants during the follow-up in 1997–2016, stratified by smoking. Adjusted for gender, BMI and socio-economic status, PAR% for smoking with a target value of no smoking was 53.6.

**Table 2:** Hazard ratios (HR) with 95% confidence intervals (CI) and population attributable risk percentages (PAR%) for the different models tested.

Covariates	HR (95% CI)	PAR%	Missing, n (%)
Model1:		55.8*	1252 (14.4)
History of regular smoking			
No	1.0		
10 pack years or fewer	2.8* (1.4–5.5)		
Over 10 pack years	6.4* (3.3–12.3)		
Model2:		55.8*	1252 (14.4)
History of regular smoking			
No	1.0		
10 pack years or fewer	2.8* (1.4–5.5)		
Over 10 pack years	6.6* (3.3–13.0)		
Gender			
Men	1.0		
Women	1.1 (0.6–1.9)		
Model3:		55.7*	1342 (15.4)
History of regular smoking			
No	1.0		
10 pack years or fewer	2.8* (1.4–5.5)		
Over 10 pack years	6.5* (3.3–12.9)		
Gender			
Men	1.0		
Women	1.1 (0.7–2.0)		
BMI			
Normal	1.0		
Overweight/Obese	1.2 (0.7–2.0)		
Model4:		53.6*	1550 (17.8)
History of regular smoking			
No	1.0		
10 pack years or fewer	2.6* (1.3–5.2)		
Over 10 pack years	5.6* (2.8–11.2)		
Gender			
Men	1.0		
Women	1.3 (0.7–2.3)		
BMI			
Normal	1.0		
Overweight/Obese	1.2 (0.7–2.0)		
Socio-economic status			
Clerical workers, entrepreneurs	1.0		
Students, retired, unemployed	1.6 (0.8–3.4)		
Manual workers, farmers	1.8 (1.0–3.3)		
*P < 0.05

## Discussion

Our study showed that smoking is associated with an increased risk for ulnar nerve entrapment, and an increase in pack years with an even more increased risk for ulnar nerve entrapment. This finding remained after adjusting for gender, BMI and socio-economic status. In our study, BMI, gender and socio-economic status did not associate with ulnar nerve entrapment.

Smoking is associated with an increased risk for ulnar neuropathies in case-control studies^13–15,17^. However, the evidence of a higher exposure affecting the risk is contradictory and has not been previously discussed in epidemiological studies^13,15,17^. So far, the only cohort study we found about smoking and ulnar neuropathies was based on a case-referent study and suggests that current smoking was associated with more severe symptoms in patients with ulnar neuropathies^10^. Compared with the findings in previous studies, our results in a large birth cohort reinforce their results. As far as we are aware, no previous studies about the association between smoking and ulnar entrapment neuropathies based on the general population exist.

The mechanism by which smoking might increase the risk for ulnar entrapment neuropathies remains unknown: both mechanical and metabolic explanations have been considered. Smoking could reduce circulation and cause fibrosis in the nerve^21–23^. Smoking a cigarette can reduce blood flow^24,25^, and chronic smoking could impair the microvascular circulation, leading to transient ischemia^26^. However, smoking posture and repetitive elbow flexion could mechanically damage the ulnar nerve, cause fibrosis and prevent its sliding in cubital tunnel^27^. Richardson and colleagues (2009) and Bartels and Verbeek (2007) showed in their studies that the preferred smoking hand, hand dominance and the side in which cubital syndrome occurred did not correlate with each other^13,15^. Although smoking posture involves elbow flexion, a mechanical explanation covering all the increased risk caused by smoking is unlikely. In our study, the side of the affected hand or preferred smoking hand were not recorded. Thus, the influence of repetitive elbow extension and trauma to the ulnar nerve at the elbow cannot be excluded in this study. The effect of smoking on other sites of ulnar nerve entrapment than the cubital tunnel remains unknown. In our study, the risk for ulnar nerve entrapment neuropathies increased as the magnitude of smoking increased. We hypothesize that this could be due increased repetitive trauma to the ulnar nerve during elbow flexion in cubital tunnel syndrome and more severe ischemia to the nerve caused by increased smoking in all ulnar entrapment neuropathies.

Ulnar entrapment neuropathies are associated with systemic disorders such as diabetes^7^, hypothyroidism^8^, renal disease^9^ in previous studies. In this cohort, only three participants diagnosed with ulnar entrapment neuropathies during the follow-up had hypothyroidism at the age of 31 years. None of the 66 participants with diagnosed ulnar nerve entrapment neuropathies during the follow-up were diagnosed with diabetes, renal disease, rheumatoid arthritis or cancer before the age of 31 years. Moreover, none of them were diagnosed with any of these conditions during the follow-up. In this cohort, these conditions were not associated with ulnar entrapment neuropathies.

Compared with previous studies^5,6^, BMI did not associate with ulnar entrapment neuropathies in our study. The participants’ BMI was calculated in 1997 when they were 31 years old. At that time, 51.0% of men and 70.0% of women in NFBC1966 had normal weight or were underweight. Since the 1990s, the prevalence of overweight and obesity have been rising in Europe^28,29^, and the participants’ mean BMI could have changed over the nearly twenty-year follow-up.

Socio-economic status did not associate with ulnar entrapment neuropathies in our study. When adjusted for all the background factors, HR for manual workers and farmers was 1.77 (reference group clerical workers and entrepreneurs), but not significant (95% CI = 0.96–3.27). Socio-economic status describes occupation and activity in working life, but does not directly reveal the biomechanical exposures encountered in daily living. Thus, our results on socio-economic status might not be directly comparable with studies on occupational biomechanical exposures affecting the risk for ulnar entrapment neuropathies.

As a conservative treatment of ulnar entrapment neuropathies is often not satisfying^1^, identifying lifestyle factors related to them is essential for prevention. PAR% for smoking varied between 53.6% and 55.8% in different models in our study, indicating that the excess risk for ulnar entrapment neuropathies caused by smoking is considerable.

To our best knowledge, this is the first birth cohort study about risk factors for ulnar nerve entrapment. NFBC1966 represents the Finnish general population very well. The participants are the same age and come from all socio-economic classes. The follow-up-time is long (18.48 years), and the sample is very large (N = 8718 in this study). The attendance rate of the 31-year study was 77.4%. We used data from the Finnish Care Register for Health Care; the data is reliable and comprehensive, as it identifies 80–99% of the common diagnoses^30^ and basically covers the whole Finnish healthcare system. The diagnoses are coded according to ICD. All ulnar entrapment neuropathies are coded under the same code in ICD, and could not be differentiated. We assume that the vast majority of ulnar nerve entrapments are cubital tunnel syndrome in our data. We used register data only from hospital polyclinics (specialist care), not health centers (primary care), because suspicion of a diagnosis is coded the same as a verified diagnosis in ICD. Using only hospital data might explain the small number of outcomes in this cohort; patients with mild symptoms and patients not willing to visit a hospital polyclinic could be considered healthy.

This study has several limitations. The baseline examination was done in 1997, and the follow-up period extended until the end of 2016. During such a long period of time, defining the causation between baseline variables and the outcome might be unreliable, as the baseline characteristics might change over time. Also, the diagnoses were not verified from patient records, and thus there is a possibility of misdiagnoses and misclassifications. As the etiology of ulnar entrapment neuropathies is not fully understood, there might be confounding factors that are not included in our analyses. The small number of outcomes, with only 66 cases, might limit statistical power of the analyses.

To conclude, our study showed that smoking is associated with a higher risk for ulnar entrapment neuropathies, accounting for a notably large proportion of the risk. To confirm these findings, further population-based studies are needed.

## Data Availability

The data that support the findings of this study are available from the Northern Finland Birth Cohorts, but restrictions apply to the availability of these data, which were used under license for the current study, and so are not publicly available. The authors have no right to share the data.
